# Impact of bronchiolitis guidelines publication on primary care prescriptions in the Italian pediatric population

**DOI:** 10.1038/s41533-021-00228-w

**Published:** 2021-03-19

**Authors:** Elisa Barbieri, Anna Cantarutti, Sara Cavagnis, Luigi Cantarutti, Eugenio Baraldi, Carlo Giaquinto, Daniele Donà

**Affiliations:** 1 Division of Paediatric Infectious Diseases, Department of Women’s and Children’s Health, University of Padova, Padua, Italy; 2 Department of Statistics and Quantitative Methods, Unit of Biostatistics Epidemiology and Public Health, University of Milano-Bicocca, Milan, Italy; 3 Pedianet Project, Padua, Italy; 4 Unit of Neonatal Intensive Care, Department of Woman’s and Child’s Health, University of Padova, Padua, Italy

**Keywords:** Paediatric research, Paediatric research

## Abstract

In Italy, two clinical practice guidelines for the diagnosis and treatment of bronchiolitis were published in October 2014 and December 2015. We evaluated prescriptions for bronchiolitis in children aged 0–24 months before (December 2012–December 2014), in between (December 2014–December 2015) and after (December 2015–December 2018) the guidelines publications. Data were retrieved from the Pedianet database; the measured outcomes were prescriptions rates of antibiotics, corticosteroids, β2-agonists, and other respiratory drugs. In 1011 out of 1581 episodes, patients received at least one treatment, with a total of 2003 prescriptions. The rate of treated bronchiolitis decreased from 66% to 57% (*p* < 0.001) after the publication of the second guideline; the highest reduction was in younger patients (from 57% to 44%, *p* = 0.013). Overall antibiotic prescriptions rate did not change, with 31.6% of the patients still receiving them. Our results confirm unnecessary non-evidence-based treatments in the primary care setting, with few changes after the guidelines publications.

## Introduction

Viral bronchiolitis is the most common lower respiratory tract infection in infants^1^ and has a heavy impact on pediatric healthcare^2,3^.

Bronchiolitis is mainly caused by Respiratory Syncytial Virus (RSV)^4^ and the diagnosis is based on medical history and clinical findings. Bronchiolitis is a self-limiting disease and lacks a specific etiological treatment, therefore management should be focused on supportive care, based on oxygen therapy and fluid supplementation^5–7^. Pharmacological treatment with bronchodilators, corticosteroids, antibiotics, and other medications has been proven to be not useful in reducing symptoms severity or duration^8–12^ and is currently not recommended in national and international guidelines^5–7,13,14^. In the Italian context, two guidelines were published: the first was published in 2014 by Baraldi et al., and was the result of the cooperation of 20 Italian pediatric scientific societies^7^. The second guideline is the Italian translation of the NICE guidelines by Cartabellotta et al., published in 2015^13^ and later recognized by the Italian guidelines society (“Società Nazionale Linee Guida”) of the Italian Institute of Health (“Istituto Superiore di Sanità”) as Good Clinical Practices.

The two guidelines have some minor differences in the diagnosis, management, and treatment of children with bronchiolitis. For instance, the document by Baraldi et al. refers to children up to 12 months, while the article by Cartabellotta et al., even though the Authors state that bronchiolitis is more common in the first year of life, considers children up to 2 years of age. Furthermore, when addressing the factors that should be considered when referring the child to secondary care, Baraldi et al. consider prematurity as GA < 37 weeks, while Cartabellotta et al. recommend the threshold of 32 weeks of GA. For what concerns the treatment of bronchiolitis, the second guideline is more strict in not recommending pharmacological therapies, while Baraldi et al. are less rigorous, allowing some exceptions, like a trial of inhaled salbutamol.

Despite these minor differences, both guidelines agree on the scarce role of pharmacological therapy because of a lack of evidence in treatment efficacy.

Unnecessary non-evidence-based treatment prescription is still very common, both in primary care, emergency department (ED) and hospital settings^15–20^; moreover, there is still considerable variability in diagnostic test utilization and disease management, potentially generating unnecessary and costly resources use^15,21,22^.

With this study, we aimed to evaluate the impact of both guidelines on clinical practice in the pediatric primary care (PPC) setting, by comparing prescriptions of antibiotics, corticosteroids, and bronchodilators before and after guidelines publication.

## Results

**Fig. 1:**
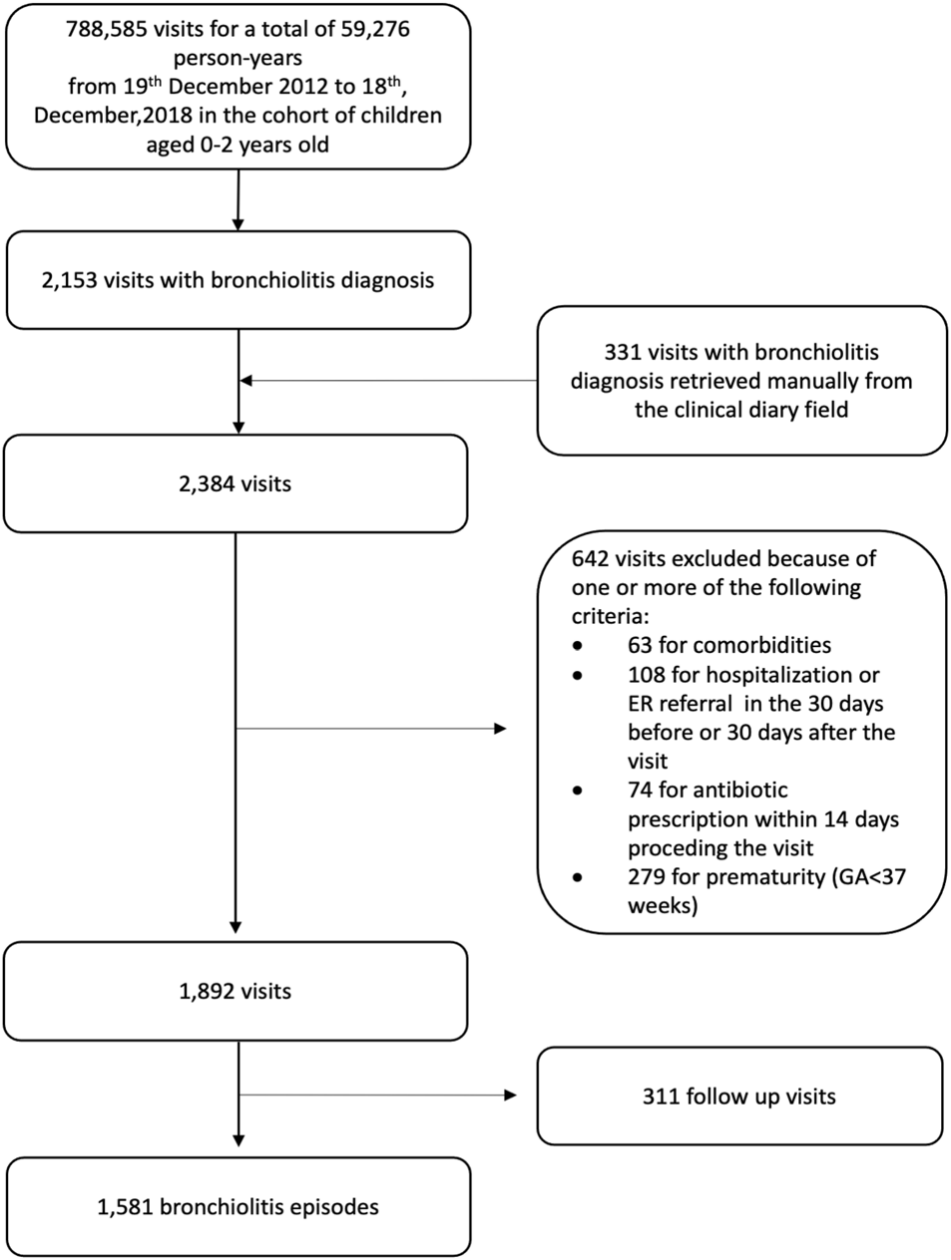
Bronchiolitis episodes’ inclusion flow chart (Pedianet, 2012–2018). ER emergency room, GA gestational age.

Complete episodes selection is described in Fig. 1.

### Population characteristics

The demographic characteristics of included patients are described in Table 1.

**Table 1:** Population characteristics in the different periods described by age group (Pedianet, 2012–2018).

	0–3 months			*p* value	4–6 months			*p* value	7–24 months			*p* value	Overall			*p* value
	Pre	Post1	Post2		Pre	Post1	Post2		Pre	Post1	Post2		Pre	Post1	Post2	
N of episodes	270	58	126		331	61	142		349	88	156		950	207	424	–
Sex, N (%), male	153 (56.7)	38 (65.5)	69 (54.8)	0.3721	192 (58.0)	46 (75.4)	87 (61.3)	0.038^1^	187 (53.6)	37 (42.0)	94 (60.3)	0.023^1^	532 (56.0)	121 (58.5)	250 (59.0)	0.542^1^
Age, Mean (SD), months	2.08 (0.84)	2.02 (0.93)	2.06 (0.85)	0.879^2^	4.98 (0.81)	5.07 (0.81)	4.96 (0.77)	0.686^2^	11.3 (4.35)	10.5 (3.46)	10.4 (3.57)	0.138^3^	6.48 (4.72)	6.54 (4.33)	6.11 (4.16)	0.316^3^
Gestational age, Mean (SD), weeks	39.1 (1.11)	39.2 (1.14)	39.1 (1.25)	0.866^2^	39.1 (1.08)	39.3 (0.85)	39.1 (1.15)	0.281^2^	39.2 (1.00)	38.9 (1.16)	39.1 (1.18)	0.042^2^	39.1 (1.06)	39.1 (1.08)	39.1 (1.19)	0.901^3^
Follow up, N (%), yes	58 (21.5)	8 (13.8)	26 (20.6)	–	47 (14.2)	9 (14.8)	27 (19.0)	–	46 (13.2)	11 (12.5)	19 (12.2)		151 (15.9)	28 (13.5)	72 (17.0)	–
Geographical location, N (%)																
South (with islands)	37 (13.7)	11 (19.0)	25 (19.8)	–	34 (10.3)	14 (23.0)	24 (16.9)	–	32 (9.2)	14 (15.9)	33 (21.2)	–	103 (10.8)	39 (18.6)	82 (19.3)	–
Centre	73 (27.0)	18 (31.0)	28 (22.2)	–	72 (21.8)	17 (27.9)	37 (26.1)	–	39 (11.2)	20 (22.7)	25 (16.0)	–	182 (19.2)	55 (26.4)	89 (21.1)	–
North	160 (59.3)	29 (50.0)	73 (57.9)	–	225 (68.0)	30 (49.2)	81 (57.0)	–	278 (79.6)	54 (61.4)	98 (62.8)	–	665 (70.0)	114 (55.0)	252 (59.5)	–

SD = standard deviation.

^1^ = Pearson χ^2^ test, ^2^ = Fisher one way ANOVA, ^3^ = Kruskall–Wallis rank sum.

Of the 1581 episodes included in the study, 454 (28.7%) patients were 0–3 months old, 534 (33.8%) were 4–6 months old and 593 (37.5%) were 7–24 months old at the time of first visits.

903 (57.1%) patients were males, and the mean age at the time of first bronchiolitis visits was around 6 months and did not differ significantly between the three periods. The majority of the patients did not have follow-up visits (84.1%) and seemed to be more frequent in younger children (20%) compared to children aged 4–6 months (15.5%) or more (12.8%).

The patients were mainly from Northern Italy (65.2%), with no changes between periods.

### Outcomes

In 1011 out of 1581 episodes included in the study, patients received at least one treatment, with a total of 2003 prescriptions.

The number of bronchiolitis treated decreased from 66.2% to 56.6% in the Pre vs Post2 period (χ^2^ test *p* < 0.001). The highest decrease was noted in children younger than 3 months (from 57.0% to 43.7%, χ^2^ test – = 0.013), while children older than 7 months did not have a significant reduction (from 70.5% to 64.7%, χ^2^ test *p* = 0.198). Complete data are shown in Supplementary Table 1.

The total number of medicines prescribed decreased significantly after the publication of the two guidelines, but still in the 56.5% of the cases in the Post2 period was prescribed at least one medicine and in the 12.7% three or more. (Supplementary Table 1).

**Fig. 2:**
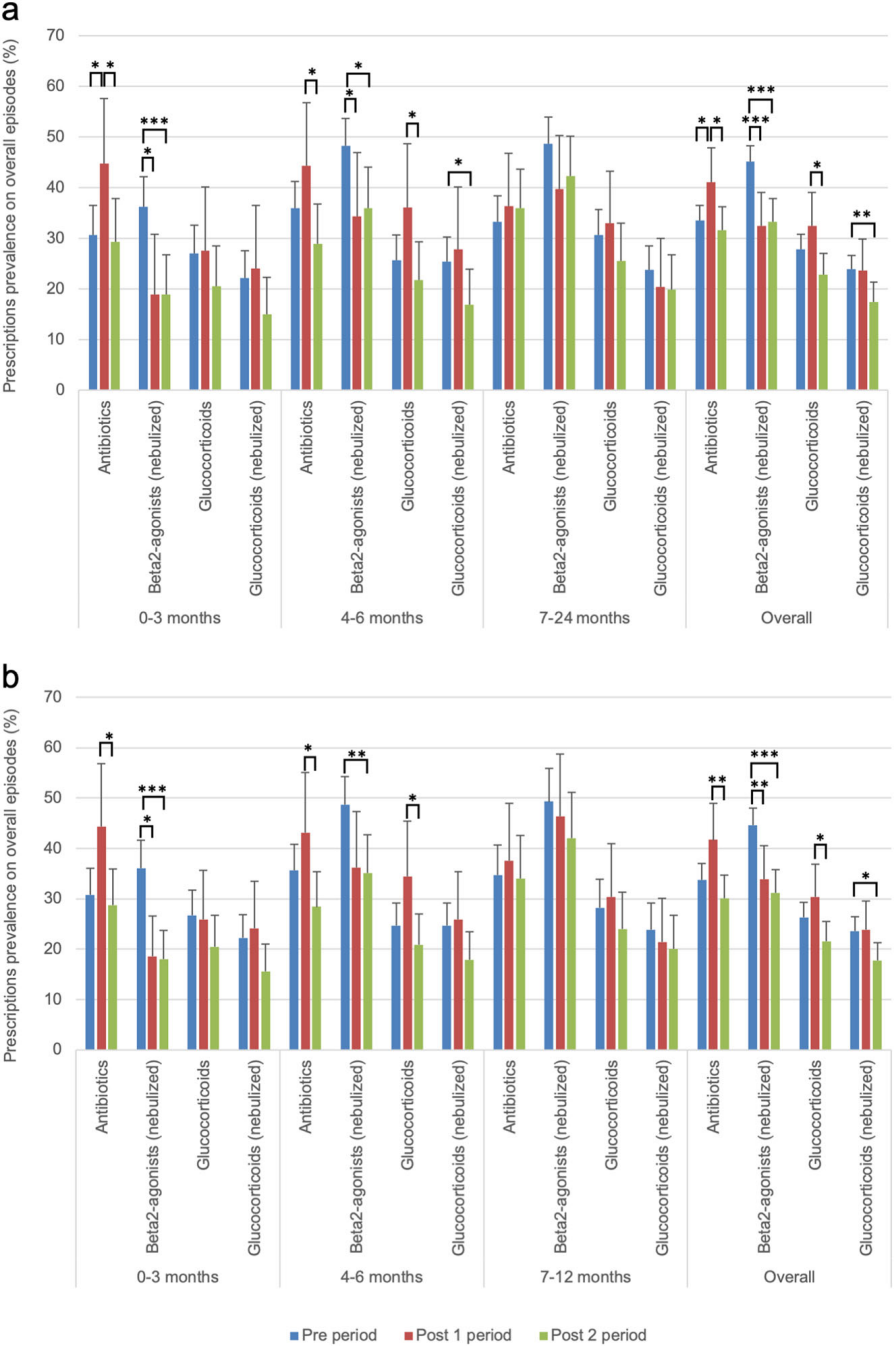
Prevalence of treatments in the different periods and age groups (Pedianet, 2012-2018). Treatments for bronchiolitis episodes in the different periods described by age group in the overall cohort (**a**) and in children < 1 year of age having a first episode of bronchiolitis (**b**). Positive error bars indicate the higher confidence interval limit (97.5%) for the percentages. Significant χ^2^ test *p* values are reported as asterisk (*), if 0.05 ≥ *p* value > 0.01 then *, if 0.01 ≥ *p* value > 0.001 then **, and if *p* value ≥ 0.001 then ***.

In total, antibiotics were prescribed in 537 episodes; in the 33.5% of episodes in the Pre period at least one antibiotic was prescribed, compared to the 41.1% and the 31.6% in the Post1 and Post2 periods respectively (Fig. 2a). Amoxicillin prescription rates increased from 11.1% to 15.6% in the Post2 period (χ^2^ test *p* = 0.019), especially in older children, varying from 9.7% to 17.9% (χ^2^ test *p* = 0.009). The prescription rates of co-amoxiclav did not change over periods, while cephalosporins prescription rates decreased significantly from 2% (Pre period) to 0.2% (Post2 period) (Fisher exact test *p* = 0.012). Macrolides prescription rates varied from 12.6% to 16.4% to 7.3% in the Pre, Post1 and Post2 periods with a significant (χ^2^ test *p* < 0.001) decrease in rates between the Post1 and Post2 periods. Regarding younger children, rates decreased significantly (χ^2^ test *p* = 0.004) from 20.7% (Post1 period) to 6.3% (Post2 period), similarly to children aged 4–6 months (from 16.4% to 7.7%, χ^2^ test *p* = 0.042) (Supplementary Table 1).

Nebulized β2-agonists’ prescriptions rates decreased relevantly in the Post1 period from 45.1% to 32.4% (χ^2^ test *p* < 0.001) with stable rates in the Post2 period (33.3%). Prescriptions rates dropped from 36.3% (Pre period) to 19.0% (Post1 and Post2 periods) in children younger than 3 months

The overall glucocorticoids prescription rates varied from 27.9% to 32.4% to 22.9% (Pre, Post1, Post2 periods). Nebulized glucocorticoids were prescribed in 23.9% of episodes in the Pre period and in the 17.5% of episodes in the Post2 period (χ^2^ test *p* = 0.008); this reduction was particularly important in children aged 0–6 months (Fig. 2a).

Other respiratory drugs were prescribed in only 21 episodes, without changes between the analyzed periods (Supplementary Table 1).

### Post hoc analysis

When considering the 1311 (82.9%) first bronchiolitis episodes in children less than 1-year old, we obtained similar results. Overall antibiotics rate did not decrease after guidelines publication, but macrolides rates dropped from 11.8% in the Pre period to 7.6% in the Post2 period (χ^2^ test *p* = 0.031). Nebulized β2-agonists’ prescriptions rates (from 44.6% to 31.2%, χ^2^ test *p* < 0.001) and nebulized glucocorticoids significantly decreased (from 23.6% to 17.7%, χ^2^ test *p* = 0.024) (Fig. 2b and Supplementary Table 2).

## Discussion

According to our results, overprescribing is still very common for patients with bronchiolitis; the unnecessary use of medicine is even more alarming in the primary care context, where children are not presenting with severe symptoms and therefore medicine use is even less justified.

In the period analyzed, we did find a decrease in prescription rates: patients were prescribed medicines in the 66% of episodes before guidelines publication and in the 57% of episodes in the periods after. Even if this reduction was significant, it is clear that there is still much room for improvement.

In particular, antibiotic prescription rates did not change at all after the publication of both guidelines, with one third of the children diagnosed with bronchiolitis receiving at least one antibiotic. Bronchiolitis has a viral etiology, hence guidelines do not recommend antibiotics as first-line treatment; moreover, it has been demonstrated that overuse of antibiotics is related to resistant bacteria strains selection. Antibiotic misuse can be ascribed to the uncertainty in diagnosing bronchiolitis: differentiating it from bacterial pneumonia represents a challenge, especially in primary care, and pediatricians tend to treat the patient for both diseases^23^. This could be improved increasing rapid test utilization in PPC, such as RSV rapid test, since infants with RSV have low probabilities of having a serious bacterial infection^24,25^, or procalcitonin and C-reactive protein rapid tests, which have been proven useful in differentiating viral and bacterial etiology in lower respiratory tract infections^26^. On the other hand, using rapid tests increase PPC-related costs, hence cost analysis is needed to determine the impact on the Italian healthcare system of extended rapid test usage.

We found a significant increase in amoxicillin prescriptions rates that could be ascribed to the publication of NICE guidelines for the treatment of pneumonia at the end of 2014, where amoxicillin was recommended as the first-line treatment for community-acquired pneumonia in children^27^. In PPC setting, IgM rapid tests or specific molecular diagnostic tests are scarcely used compared to the ED and hospital setting, thus the bacterial etiological suspect, once born, is rarely discarded. It should also be considered that the third dose to complete the recommended pneumococcal and Hib vaccination is delivered to the child at 11–12 months of age, thus family pediatricians might choose to prescribe a treatment covering a possible bacterial infection to younger children.

Another relevant finding was that children older than 7 months received more medicines than younger patients: after the publication of both bronchiolitis guidelines, in the 64.7% of episodes in children aged 7–24 months patients received pharmacological treatment, compared to the 51.9% in patients <6 months of age. This finding was coherent with the literature^28^; this difference could be explained by the fact that younger patients’ bronchiolitis usually adheres better to the diagnostic criteria, while older patients are often diagnosed with bronchiolitis but treated for recurrent wheezing. However, when considering only the first bronchiolitis episode per child (thus excluding patients treated for recurrent wheezing), patients aged 7–24 months received more frequently a pharmacological treatment.

Numerous studies conducted mainly in the hospital setting^29–38^ evaluating the impact of bronchiolitis guidelines on medicine prescriptions reported that antibiotic prescription rates were the ones with a minor decrease among all treatments.

Focusing on the PPC, two studies, conducted in the same area of Spain, were particularly relevant: the first study reported a decrease in pharmacological treatment rates (from 72.5% to 52.1%) after a quality improvement initiative including interactive informal sessions, the distribution through the mail system of an evidence-based protocol and poster display in the ambulatory room to remind bronchiolitis diagnostic criteria and treatment recommendations^29^. In the second study, quality improvement initiatives were implemented both in PPC and in the corresponding hospital ED; the strategy included interactive educational sessions, bronchiolitis guideline algorithm distributed via emails, badges with the slogan “Bronchiolitis, less is more”, informative posters in the waiting room, messages in report templates associated with the diagnostic code and monthly reports of performance^39^. With this intervention, they managed to reduce drastically the prescription of salbutamol (from 38.3% to 15.9%), antibiotics (from 29.6% to 9.5%) and corticosteroids (from 12.9% to 3.5%). The Authors ascribe these results mainly to the collaboration between ED and family pediatricians; furthermore, having the project goal visible (i.e. in posters and badges) and giving the clinicians feedback on their prescription rates might have played a role in reducing the overuse of medications.

In another study conducted in a PPC clinic in the USA, the authors reported a significant reduction in beta2-agonists prescriptions rates in children with bronchiolitis from 45.7% to 13.7%^40^. They combined guideline education for clinicians with a behavioral intervention approach based on findings revealing that being required to justify one’s action causes more self-reflection, and that individuals prefer to conform to the actions of their peers^41,42^.

The reason for prescribing non-evidence-based treatments in acute bronchiolitis may vary: according to a British study, half of the general practitioners prescribed a treatment for a potential differential diagnosis, one third because a believed efficacy in the treatment and 31% because they believe that parents expect a prescription or that the prescription removes the need for a discussion with parents (it was possible for the clinician to choose more than one reason for prescribing medication)^43^. While the first reason could be valid—since it is challenging to differentiate between a clinical diagnosis of bronchiolitis, bronchopneumonia and recurrent wheeze—the others should raise some concerns because demonstrate a potential gap in the knowledge of bronchiolitis treatment, and a supposed negative influence of parents on clinical decisions.

This study is particularly relevant because it is the first study assessing prescriptions for bronchiolitis conducted in the Italian PPC setting and one of the few studies describing the impact of bronchiolitis guidelines on non-evidence-based treatments.

The strength of our study is its size, generalizability, and representative coverage of pediatric patients. In this study, 75% of the population referring to family pediatricians enrolled in Pedianet provided consent. In Italy, it is mandatory to be enrolled in primary care and children are assigned to their family pediatrician based on their home proximity to the family pediatrician ambulatory. Following this consideration, we have no evidence supporting a selection bias.

A limitation lies in its retrospective nature. It should not be excluded that at least a few bronchiolitis cases were seen in an ED without being reported to the family pediatricians. However, those would very likely have been identified because a follow-up examination by the family pediatrician is very always recommended after ED discharge, especially for younger children. Second, as per post hoc analysis, bronchiolitis diagnosis decreased in the years, possibly due to reduced misclassification. Indeed, the first part of both guidelines implemented was dedicated to the diagnosis, focusing on signs and symptoms. Bronchiolitis diagnoses were based on clinical evaluation, including those retrieved using International Classification of Diseases, Ninth Revision, Clinical Modification (ICD9-CM) codes assigned by the family pediatrician when reporting the physical evaluation in the patient’s electronic medical chart in the software. Even if the dataset was manually validated, the impossibility to confirm clinical assessment is a well-recognized limitation in working with real-world data because it may be subjective to the attending clinician. We addressed this limit in case selection, applying an algorithm based on symptoms to the dataset. Third, it was not possible to evaluate prescription trend with a more sophisticated method such as regression analysis of interrupted time series because of the small sample size in monthly data for each outcome. Fourth, we chose to study just non-premature children with no comorbidities because of the regional differences in the reimbursement of palivizumab, a monoclonal antibody to prevent RSV infections, in respect of infants gestational age and comorbidities. Lastly, the impact of the guidelines was based on prescriptions and not on pharmacy dispensations, thus it was not possible to assess neither the “wait and see” approach (i.e. clinician could recommend the parents to wait 24–48 h before giving any medication to the child to allow the symptoms to improve spontaneously) neither the non-adherence.

In Italy, guidelines are mainly sponsored by scientific societies by publication on national and international peer-reviewed journals and emails. To change clinical practice, it is not enough for a guideline to be simply published and sent to health professionals: quality improvement represents valid strategies to decrease inappropriate prescribing^44^. The first step in this direction is represented by the campaign Choosing wisely, supported by the Italian Federation of Family Pediatricians that is implemented through clinicians’ education and development of patient-friendly material about non-evidence-based treatments, with the slogan “Doing more does not mean doing better”^45^.

Stewardship policy implementation has also proved to make a difference in reducing inappropriate prescribing. In a setting where the main challenges are represented by high patient turnover rates and diagnostic uncertainty in empiric prescription, clinical pathways, a one-page decision support algorithm summarizing the guidelines, could be a valid tool to assist prescribers in defining the diagnosis and the most appropriate treatment ^46,47^.

Non-evidence-based overprescribing of antibiotics and nebulized medication for infant bronchiolitis is still common.

For future research, we recommend a quality improvement strategy, targeting family pediatricians and ED clinicians and involving the same in the planning of the intervention, thus reducing in a more decisive way overtreatment and improving patients’ outcomes.

## Methods

### Study design

**Fig. 3:**
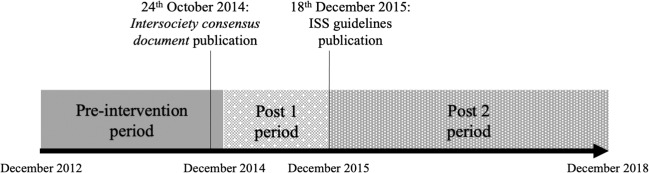
Study design (Pedianet, 2012–2018). The arrow represents the timeline where are indicated the date of the publication of the two guidelines and the study periods with the corresponding starting and ending date colored with different textures of gray. ISS Istituto Superiore di Sanita’ - Italian Institute of Health.

In this observational, uncontrolled before-after study, we assessed the changes in prescriptions for bronchiolitis in Italian PPC before (Pre period: December 2012–December 2014), in-between (Post1 period: December 2014–December 2015) and after (Post2 period: December 2015–December 2018) the publication of the two guidelines. The study flow chart is shown in Fig. 3.

### Data source

Patients have been selected to review medical records collected by family pediatricians in the Pedianet Database. Pedianet is a PPC research database that collects information on children visited by 134 family pediatricians throughout Italy. The system is based on the transmission of specific data from computerized clinical files, that the pediatricians fill out during their daily clinical practice; informed consent is required from the parents^48^. Such data, generated using common software (JuniorBit^®^), are collected anonymously in a centralized database in Padua. The database contains several types of information, such as reason for the visit, medical examination, diagnosis, health status, prescriptions, specialist’s referrals, hospitalizations, diagnostic procedures, growth parameters, and outcome data.

For this study data related to 150,018 children having at least 2 visits in the study period for a total of 2,958,459 visits were considered. Each family pediatrician had a median of 1156 (Interquartile range: 856–1886) patients registered in Pedianet in the study period with 54.5% of them residing in the North of Italy (Friuli-Venezia Giulia, Liguria, Lombardia, Piemonte, Veneto) and the rest in the Centre (15.7%, Lazio, Marche, Toscana) and in the South or Islands (29.9%, Abruzzo, Campania, Sardegna, Sicilia) according to ISTAT areas^49^.

The study and the access to the database were approved by the Internal Scientific Committee of Pedianet.

### Study population and case identification

We included all children aged 0–24 months who were registered with one of the Pedianet family pediatricians since the birth between December 2012 and December 2018 and for whom parental informed consent was provided, with a diagnosis of acute bronchiolitis.

Cases have initially been identified from coded diagnosis of acute bronchiolitis (ICD9-CM codes 466.1, 466.11 or 466.19) and then from descriptive diagnosis in the free text fields. We also searched for patients with symptoms that can be ascribed to bronchiolitis; a case of acute bronchiolitis was defined as a first episode of respiratory distress combined with at least two of the following symptoms: coryza, cough, wheezing or crackles, tachypnea, chest retractions, skin color changes, nasal flaring, and fever^7,13^. All the potential episodes were manually validated by two independent researchers (EB and SC) to exclude any false-positive cases. In case of disagreements, a consensus was reached with an expert pediatrician (DD).

In order to avoid duplicates, all visits occurring within 30 days of the initial diagnosis were considered as follow-up visits.

We considered the following as exclusion criteria: chronic complex conditions (such as cystic fibrosis, diabetes, chronic obstructive pulmonary disease), immunodeficiency or immunosuppressive therapy, prematurity (<37 weeks of gestational age), Down syndrome, congenital heart disease other than small ventricular septal defect, hospitalization or visit in the ED in the 30 days before or after the diagnosis, concomitant bacterial infection (i.e. acute otitis media or pharyngotonsillitis) or ongoing antibiotic therapy (defined as an antibiotic prescription in the 14 days before the bronchiolitis case) and previous admission for wheeze to the ED.

### Outcomes

The measured outcomes were the rates of prescriptions of oral systemic antibiotics, oral corticosteroids and nebulized corticosteroids, nebulized β2-agonists, and other respiratory drugs identified with Anatomical Therapeutic Chemical codes (Supplementary Table 3).

We considered only the first prescription per drug class linked to the specific bronchiolitis episode.

### Statistical analysis

Data analysis was performed on the whole cohort and then stratified based on age class (0–3, 4–6, and 7–24 months of age) basing the stratification in accordance with guidelines treatment indication. Demographic variables were compared among all periods and outcome variables were compared against all periods (Pre vs Post1, Pre vs Post2, and Post1 vs Post2). Post hoc analysis was performed considering just the first bronchiolitis episode per child and limiting the analysis to patient <1 year of age.

Categorical variables were expressed using frequencies and percentages; associations between these variables were assessed using the χ^2^ test (Pearson or Fisher exact test where appropriate).

Continuous variables were expressed as means and standard deviation (SD); these variables were compared using the appropriate non-paired test after assessing for the normality (Fisher one-way ANOVA or Kruskall–Wallis rank sum). The 95% confidence intervals were calculated with the Wilson Score method.

The analysis was performed using R statistical software—v. 3.6.2 (Vienna, Austria)^50^. Two sided *p* < 0.05 was considered statistically significant.

### Reporting summary

Further information on research design is available in the Nature Research Reporting Summary linked to this article.

## Supplementary information

Supplementary Material

Reporting Summary

## Data Availability

The data used in this study cannot be made available in the manuscript, the supplemental files or in a public repository due to Italian data protection laws. The anonymized datasets generated during and/or analyzed during the current study can be provided on reasonable request, from the corresponding author, after written approval by the Internal Scientific Committee (info@pedianet.it).

## Code availability

Available upon request.
