# Supplementary material for: Impact of bronchiolitis guidelines publication on primary care prescriptions in the Italian pediatric population
Source: NPJ Prim Care Respir Med. 2021 Mar 19;31:15. doi: 10.1038/s41533-021-00228-w (PMC7979748; doi:10.1038/s41533-021-00228-w)
Supplement: Supplementary file 1 — Supplementary Material [file 41533_2021_228_MOESM1_ESM.pdf]

Supplementary material

Tables

Supplementary Table 1. Treatments for bronchiolitis in the different periods described by age group. Significant χ^2 test p values ≤ 0.05 are reported (Pedianet, 2012-2018).

Supplementary Table 2. Treatments for the first episodes of bronchiolitis in the different periods described by age group in children < 1 year of age. Significant χ^2 test p values ≤ 0.05 are reported (Pedianet, 2012-2018).

Supplementary Table 3. ATC selection codes

Supplementary Table 1. Treatments for bronchiolitis in the different periods described by age group. Significant χ^2 test p values ≤ 0.05 are reported (Pedianet, 2012-2018).

	Pre	Post1	Post2	First guideline (pre vs post1) p value	Second guideline (pre vs post2) p value	Control (post1 vs post2) p value
0-3 months						
N of episodes	270	58	126			
Treatment, N (%), Yes	154 (57.0)	34 (58.6)	55 (43.7)		0.013	
Antibiotics, N (%)	83 (30.7)	26 (44.8)	37 (29.4)	0.039		0.04
Amoxicillin, N (%)	29 (10.7)	6 (10.3)	17 (13.5)			
Co-amoxiclav, N (%)	15 (5.6)	7 (12.1)	12 (9.5)			
Cephalosporins, N (%)	6 (2.2)	1 (1.7)	0 (.)			
Macrolides, N (%)	33 (12.2)	12 (20.7)	8 (6.3)			0.004
Other antibiotics, N (%)	0 (.)	0 (.)	0 (.)			
Beta2-agonists (nebulized), N (%)	98 (36.3)	11 (19.0)	24 (19.0)	0.011	<0.001	
Glucocorticoids, N (%)	73 (27.0)	16 (27.6)	26 (20.6)			
Glucocorticoids (nebulized), N (%)	60 (22.2)	14 (24.1)	19 (15.1)			
Other respiratory drugs, N (%)	3 (1.1)	1 (1.7)	0 (.)			
4-6 months						
N of episodes	331	61	142			
Treatment, N (%), Yes	229 (69.2)	45 (73.8)	84 (59.2)		0.035	0.047
Antibiotics, N (%)	119 (36.0)	27 (44.3)	41 (28.9)			0.033
Amoxicillin, N (%)	42 (12.7)	10 (16.4)	21 (14.8)			
Co-amoxiclav, N (%)	29 (8.8)	7 (11.5)	8 (5.6)			
Cephalosporins, N (%)	6 (1.8)	1 (1.6)	0 (.)			
Macrolides, N (%)	48 (14.5)	10 (16.4)	11 (7.7)		0.042	
Other antibiotics, N (%)	2 (0.6)	0 (.)	1 (0.7)			
Beta2-agonists (nebulized), N (%)	160 (48.3)	21 (34.4)	51 (35.9)	0.045	0.013	
Glucocorticoids, N (%)	85 (25.7)	22 (36.1)	31 (21.8)			0.034
Glucocorticoids (nebulized), N (%)	84 (25.4)	17 (27.9)	24 (16.9)		0.044	
Other respiratory drugs, N (%)	2 (0.6)	0 (.)	1 (0.7)			
7-24 months						
N of episodes	349	88	156			
Treatment, N (%), Yes	246 (70.5)	63 (71.6)	101 (64.7)			
Antibiotics, N (%)	116 (33.2)	32 (36.4)	56 (35.9)			
Amoxicillin, N (%)	34 (9.7)	13 (14.8)	28 (17.9)		0.009	
Co-amoxiclav, N (%)	38 (10.9)	6 (6.8)	16 (10.3)			
Cephalosporins, N (%)	7 (2.0)	1 (1.1)	1 (0.6)			
Macrolides, N (%)	39 (11.2)	12 (13.6)	12 (7.7)			
Other antibiotics, N (%)	0 (.)	0 (.)	0 (.)			
Beta2-agonists (nebulized), N (%)	170 (48.7)	35 (39.8)	66 (42.3)			
Glucocorticoids, N (%)	107 (30.7)	29 (33.0)	40 (25.6)			
Glucocorticoids (nebulized), N (%)	83 (23.8)	18 (20.5)	31 (19.9)			
Other respiratory drugs, N (%)	10 (2.9)	2 (2.3)	2 (1.3)			
Overall						
N of episodes	950	207	424			
Treatment, N (%), Yes	629 (66.2)	142 (68.6)	240 (56.6)		<0.001	0.004
Antibiotics, N (%)	318 (33.5)	85 (41.1)	134 (31.6)	0.038		0.019
Amoxicillin, N (%)	105 (11.1)	29 (14.0)	66 (15.6)		0.019	
Co-amoxiclav, N (%)	82 (8.6)	20 (9.7)	36 (8.5)			
Cephalosporins, N (%)	19 (2.0)	3 (1.4)	1 (0.2)		0.012[1]	
Macrolides, N (%)	120 (12.6)	34 (16.4)	31 (7.3)		0.004	<0.001
Other antibiotics, N (%)	2 (0.2)	0 (.)	1 (0.2)			
Beta2-agonists (nebulized), N (%)	428 (45.1)	67 (32.4)	141 (33.3)	<0.001	<0.001	
Glucocorticoids, N (%)	265 (27.9)	67 (32.4)	97 (22.9)			0.011
Glucocorticoids (nebulized), N (%)	227 (23.9)	49 (23.7)	74 (17.5)		0.008	
Other respiratory drugs, N (%)	15 (1.6)	3 (1.4)	3 (0.7)			

[1] = Fisher exact test

Supplementary Table 2. Treatments for the first episodes of bronchiolitis in the different periods described by age group in children < 1 year of age. Significant χ^2 test p values ≤ 0.05 are reported (Pedianet, 2012-2018).

	Pre	Post1	Post2	First guideline (pre vs post1) p value	Second guideline (pre vs post2) p value	Control (post1 vs post2) p value
0-3 months						
N of episodes	266	54	122			
Treatment, N (%), Yes	150 (56.4)	31 (57.4)	52 (42.6)		0.012	
Antibiotics, N (%)	82 (30.8)	24 (44.4)	35 (28.7)			0.041
Amoxicillin, N (%)	29 (10.9)	6 (11.1)	15 (12.3)			
Co-amoxiclav, N (%)	15 (5.6)	6 (11.1)	12 (9.8)			
Cephalosporins, N (%)	6 (2.3)	0 (0)	0 (0)			
Macrolides, N (%)	32 (12.0)	12 (22.2)	8 (6.6)	0.047		0.003
Other antibiotics, N (%)	0 (0)	0 (0)	0 (0)			
Beta2-agonists (nebulized), N (%)	96 (36.1)	10 (18.5)	22 (18.0)	0.012	< 0.001	
Glucocorticoids, N (%)	71 (26.7)	14 (25.9)	25 (20.5)			
Glucocorticoids (nebulized), N (%)	59 (22.2)	13 (24.1)	19 (15.6)			
Other respiratory drugs, N (%)	3 (1.1)	0 (0)	0 (0)			
4-6 months						
N of episodes	308	58	134			
Treatment, N (%), Yes	214 (69.5)	42 (72.4)	78 (58.2)		0.021	
Antibiotics, N (%)	110 (35.7)	25 (43.1)	38 (28.4)			0.046
Amoxicillin, N (%)	38 (12.3)	9 (15.5)	20 (14.9)			
Co-amoxiclav, N (%)	27 (8.8)	7 (12.1)	7 (5.2)			
Cephalosporins, N (%)	5 (1.6)	1 (1.7)	0 (0)			
Macrolides, N (%)	45 (14.6)	9 (15.5)	10 (7.5)		0.036	
Other antibiotics, N (%)	2 (0.6)	0 (0)	1 (0.7)			
Beta2-agonists (nebulized), N (%)	150 (48.7)	21 (36.2)	47 (35.1)		0.008	
Glucocorticoids, N (%)	76 (24.7)	20 (34.5)	28 (20.9)			0.046
Glucocorticoids (nebulized), N (%)	76 (24.7)	15 (25.9)	24 (17.9)			
Other respiratory drugs, N (%)	1 (0.3)	0 (0)	1 (0.7)			
7-12 months						
N of episodes	213	56	100			
Treatment, N (%), Yes	149 (70.0)	40 (71.4)	61 (61.0)			
Antibiotics, N (%)	74 (34.7)	21 (37.5)	34 (34.0)			
Amoxicillin, N (%)	27 (12.7)	8 (14.3)	17 (17.0)			
Co-amoxiclav, N (%)	29 (13.6)	4 (7.1)	9 (9.0)			
Cephalosporins, N (%)	3 (1.4)	0 (0)	0 (0)			
Macrolides, N (%)	16 (7.5)	9 (16.1)	9 (9.0)	0.050		
Other antibiotics, N (%)	0 (0)	0 (0)	0 (0)			
Beta2-agonists (nebulized), N (%)	105 (49.3)	26 (46.4)	42 (42.0)			
Glucocorticoids, N (%)	60 (28.2)	17 (30.4)	24 (24.0)			
Glucocorticoids (nebulized), N (%)	51 (23.9)	12 (21.4)	20 (20.0)			
Other respiratory drugs, N (%)	2 (0.9)	2 (3.6)	1 (1.0)			
Overall						
N of episodes	787	168	356			
Treatment, N (%), Yes	513 (65.2)	113 (67.3)	191 (53.7)		< 0.001	0.003
Antibiotics, N (%)	266 (33.8)	70 (41.7)	107 (30.1)			0.009
Amoxicillin, N (%)	94 (11.9)	23 (13.7)	52 (14.6)			
Co-amoxiclav, N (%)	71 (9.0)	17 (10.1)	28 (7.9)			
Cephalosporins, N (%)	14 (1.8)	1 (0.6)	0 (0)		0.007[1]	
Macrolides, N (%)	93 (11.8)	30 (17.9)	27 (7.6)	0.034	0.031	< 0.001
Other antibiotics, N (%)	2 (0.3)	0 (0)	1 (0.3)			
Beta2-agonists (nebulized), N (%)	351 (44.6)	57 (33.9)	111 (31.2)	0.011	< 0.001	
Glucocorticoids, N (%)	207 (26.3)	51 (30.4)	77 (21.6)			0.030
Glucocorticoids (nebulized), N (%)	186 (23.6)	40 (23.8)	63 (17.7)		0.024	
Other respiratory drugs, N (%)	6 (0.8)	2 (1.2)	2 (0.6)			

[1] = Fisher exact test

Supplementary Table 3. ATC selection codes

Group	ATC
Antibiotics	J01xxx
Amoxicillin	J01CA04
Amoxicillin and clavulanic acid	J01CR02
Cephalosporins	J01Dxx
Macrolides	J01FAxx
Other antibiotics	J01xxx not included in the previous categories
Beta2-agonists (nebulized)	R03Axx
Glucocorticoids	H02Axx
Glucocorticoids (nebulized)	R03BAxx
Other respiratory drugs	R03xxx
