# Supplementary material for: Impact of bronchiolitis guidelines publication on primary care prescriptions in the Italian pediatric population
Source: NPJ Prim Care Respir Med. 2021 Mar 19;31:15. doi: 10.1038/s41533-021-00228-w (PMC7979748; doi:10.1038/s41533-021-00228-w)
Supplement: Supplementary file 2 — Reporting Summary [file 41533_2021_228_MOESM2_ESM.pdf]

nature research

Corresponding author(s): Elisa Barbieri

Last updated by author(s): Feb 2, 2021

Reporting Summary

Nature Research wishes to improve the reproducibility of the work that we publish. This form provides structure for consistency and transparency in reporting. For further information on Nature Research policies, see our Editorial Policies and the Editorial Policy Checklist.

Statistics

For all statistical analyses, confirm that the following items are present in the figure legend, table legend, main text, or Methods section.

n/a	Confirmed	
☐	☒	The exact sample size (*n*) for each experimental group/condition, given as a discrete number and unit of measurement
☒	☐	A statement on whether measurements were taken from distinct samples or whether the same sample was measured repeatedly
☐	☒	The statistical test(s) used AND whether they are one- or two-sided<br>*Only common tests should be described solely by name; describe more complex techniques in the Methods section.*
☒	☐	A description of all covariates tested
☒	☐	A description of any assumptions or corrections, such as tests of normality and adjustment for multiple comparisons
☐	☒	A full description of the statistical parameters including central tendency (e.g. means) or other basic estimates (e.g. regression coefficient) AND variation (e.g. standard deviation) or associated estimates of uncertainty (e.g. confidence intervals)
☐	☒	For null hypothesis testing, the test statistic (e.g. *F*, *t*, *r*) with confidence intervals, effect sizes, degrees of freedom and *P* value noted<br>*Give P values as exact values whenever suitable.*
☒	☐	For Bayesian analysis, information on the choice of priors and Markov chain Monte Carlo settings
☒	☐	For hierarchical and complex designs, identification of the appropriate level for tests and full reporting of outcomes
☒	☐	Estimates of effect sizes (e.g. Cohen's *d*, Pearson's *r*), indicating how they were calculated

Our web collection on statistics for biologists contains articles on many of the points above.

Software and code

Policy information about availability of computer code

Data collection	Patients have been selected reviewing medical records collected by primary care pediatricians in the Pedianet Database. Pedianet is a PPC research database that collects information on children visited by 134 family pediatricians throughout Italy. The system is based on the transmission of specific data from computerized clinical files, that the pediatricians fill out during their daily clinical practice; informed consent is required from the parents . Such data, generated using common software (JuniorBit ®), are collected anonymously in a centralized database in Padua. The database contains several types of information, such as reason for the visit, medical examination, diagnosis, health status, prescriptions, specialist's referrals, hospitalizations, diagnostic procedures, growth parameters and outcome data.
Data analysis	The analysis were performed using R statistical software – v. 3.6.2 (Vienna, Austria).

For manuscripts utilizing custom algorithms or software that are central to the research but not yet described in published literature, software must be made available to editors and reviewers. We strongly encourage code deposition in a community repository (e.g. GitHub). See the Nature Research guidelines for submitting code & software for further information.

Data

Policy information about availability of data

All manuscripts must include a data availability statement. This statement should provide the following information, where applicable:

- Accession codes, unique identifiers, or web links for publicly available datasets
- A list of figures that have associated raw data
- A description of any restrictions on data availability

The data used in this study cannot be made available in the manuscript, the supplemental files or in a public repository due to Italian data protection laws. The anonymized datasets generated during and/or analyzed during the current study can be provided on reasonable request, from the corresponding author, after written approval by the Internal Scientific Committee (info@pedianet.it).

Field-specific reporting

Please select the one below that is the best fit for your research. If you are not sure, read the appropriate sections before making your selection.

☐ Life sciences ☒ Behavioural & social sciences ☐ Ecological, evolutionary & environmental sciences

For a reference copy of the document with all sections, see nature.com/documents/nr-reporting-summary-flat.pdf

Behavioural & social sciences study design

All studies must disclose on these points even when the disclosure is negative.

Study description	This is an observational, uncontrolled before-after study
Research sample	We included in the study all children aged 0 to 24 months who were registered with one of the Pedianet family pediatricians since the birth between December 2012 and December 2018 and were diagnosed with acute bronchiolitis.
Sampling strategy	No sample size calculation was performed
Data collection	Patients have been selected reviewing medical records collected by primary care pediatricians in the Pedianet Database. Pedianet is a PPC research database that collects information on children visited by 134 family pediatricians throughout Italy. The system is based on the transmission of specific data from computerized clinical files, that the pediatricians fill out during their daily clinical practice; informed consent is required from the parents. Such data, generated using common software (JuniorBit ®), are collected anonymously in a centralized database in Padua. The database contains several types of information, such as reason for the visit, medical examination, diagnosis, health status, prescriptions, specialist's referrals, hospitalizations, diagnostic procedures, growth parameters and outcome data. Cases have initially been identified from coded diagnosis of acute bronchiolitis (International Classification of Diseases, Ninth Revision, Clinical Modification codes 466.1, 466.11 or 466.19) and then from descriptive diagnosis in the free text fields. We also searched for patients with symptoms that can be ascribed to bronchiolitis; a case of acute bronchiolitis was defined as a first episode of respiratory distress combined with at least two of the following symptoms: coryza, cough, wheezing or crackles, tachypnea, chest retractions, skin color changes, nasal flaring and fever. All the potential episodes were manually validated by two independent researchers (EB and SC) to exclude any false-positive cases. In case of disagreements, a consensus was reached with an expert pediatrician (DD).
Timing	There is no gap between collection period. i.e.Pre period: December 2012- December 2014, Post1 period: December 2014-December 2015, Post2 period: December 2015-December 2018
Data exclusions	We considered the following as exclusion criteria: chronic complex conditions (such as cystic fibrosis, diabetes, chronic obstructive pulmonary disease), immunodeficiency or immunosuppressive therapy, prematurity (< 37 weeks of gestational age), Down syndrome, congenital heart disease other than small ventricular septal defect, hospitalization in the 30 days before or after the diagnosis, concomitant bacterial infection (i.e. acute otitis media or pharyngotonsillitis) or ongoing antibiotic therapy (defined as antibiotic prescription in the 14 days before the bronchiolitis case) and previous admission for wheeze to the ED.
Non-participation	This section is not applicable. We included only patients continuosly registered in the database since birth.
Randomization	This section is not applicable.

Reporting for specific materials, systems and methods

We require information from authors about some types of materials, experimental systems and methods used in many studies. Here, indicate whether each material, system or method listed is relevant to your study. If you are not sure if a list item applies to your research, read the appropriate section before selecting a response.

Materials & experimental systems

n/a	Involved in the study	
☒	☐	Antibodies
☒	☐	Eukaryotic cell lines
☒	☐	Palaeontology and archaeology
☒	☐	Animals and other organisms
☐	☒	Human research participants
☒	☐	Clinical data
☒	☐	Dual use research of concern

Methods

n/a	Involved in the study	
☒	☐	ChIP-seq
☒	☐	Flow cytometry
☒	☐	MRI-based neuroimaging

Human research participants

Policy information about studies involving human research participants

Population characteristics	See above
Recruitment	Family paediatrician that are partecipating in Pedianet ask the parents the consent for the use of their child's demographic and clinical data after anonimization. The parents must sign a written consent.
Ethics oversight	The study and the access to the database were approved by the Internal Scientific Committee of Pedianet.

Note that full information on the approval of the study protocol must also be provided in the manuscript.
